# Construction, molecular characterization, and safety assessment of *purB* mutant of *Salmonella* Gallinarum

**DOI:** 10.3389/fmicb.2024.1467230

**Published:** 2024-11-13

**Authors:** Masham Mukhtar, Aamir Ghafoor, Michael McClelland, Fareeha Akhtar, Muhammad Adil Rasheed

**Affiliations:** ^1^University Diagnostic Laboratory, Institute of Microbiology, University of Veterinary and Animal Science, Lahore, Pakistan; ^2^Department of Microbiology and Molecular Genetics, University of California, Irvine, Irvine, CA, United States; ^3^Department of Pharmacology and Toxicology, University of Veterinary and Animal Sciences, Lahore, Pakistan

**Keywords:** *Salmonella enterica* serovar Gallinarum, purine biosynthesis, *purB* gene, virulence, safety

## Abstract

This study involves the development and molecular characterization of the isogenic markerless knockout mutant SG Δ*purB*, a genetically engineered live attenuated strain aimed at controlling *Salmonella* Gallinarum (SG) infection in poultry. The mutant was generated by deleting the *purB* gene using *λ*-Red recombination technology, impairing adenylosuccinate lyase, necessary for purine biosynthesis. An 1,180 bp deletion was engineered within the *purB* gene, leaving a residual 298 bp genomic scar resulting in a purine auxotrophic mutant. Phenotypically, SG Δ*purB* showed a 66.5% reduction in growth in LB broth compared to the wild-type strain and failed to grow in minimal media without adenosine. Growth was restored to near wild-type levels with 0.3 mM adenosine supplementation, demonstrating the strain’s conditional attenuation. *In vivo* pathogenicity assessments revealed that oral inoculation of SG Δ*purB* into 3-day-old chickens at a dose of 2 × 10^8^ CFU resulted in zero mortality, compared to an 80% mortality rate in chickens challenged with the wild-type strain. The SG Δ*purB* strain exhibited significantly reduced clinical signs and lesion scores, with clinical sign scores dropping from 2.5/3 with the wild-type to 0.4/3 with the Δ*purB* mutant, and lesion scores decreasing from 2.9/3 to 0.3/3. Additionally, the mutant was efficiently cleared from liver and spleen tissues by 14 days post-inoculation, unlike the wild-type strain, which persisted until the experiment’s end on day 21. The SG Δ*purB* mutant shows potential as a safe alternative for preventing fowl typhoid, highlighting the promise of targeted genetic attenuation in developing effective vaccines for poultry diseases.

## 1 Introduction

Salmonellosis continues to be a prevalent infectious disease within the poultry sector globally (Calnek, 1991; Chappell et al., 2009). *Salmonella enterica* serovar Gallinarum (SG), a host-specific serovar., induces fowl typhoid (FT), a systemic condition characterized by septicemia affecting domestic poultry of all ages, including week-old chicks (Calnek, 1991; Kwon et al., 2010). Fowl typhoid persists as a significant concern in various regions experiencing elevated ambient temperatures, which complicate environmental hygiene, leading to considerable economic losses due to mortality, morbidity, and reduced egg production (Zhang-Barber et al., 1998; Berchieri et al., 2001; Kwon et al., 2010). Vaccination represents an effective approach for the prevention of *Salmonella* infections (Mastroeni et al., 2001). Live attenuated *Salmonella* strains are more effective as vaccines against salmonellosis in various animal species compared to inactivated vaccines (Smith, 1956). Inactivated vaccines stimulate antibody production but do not significantly enhance the cellular immunity (Gast et al., 1993). In contrast, live vaccines generate strong humoral and cellular immune responses, particularly when the vaccine strain is invasive (Babu et al., 2003). Despite not having full accreditation, the widely recognized live vaccine strain 9R has substantially contributed to reducing the prevalence of the disease (Smith, 1956; Bouzoubaa et al., 1989; Kwon et al., 2010), However, the persistence of residual virulence, incomplete immunity, and the undefined genotype of the 9R vaccine strain have prompted researchers to pursue the development of improved vaccine strains (Griffin and Barrow, 1993; Shah et al., 2007; Matsuda et al., 2010; Penha Filho et al., 2010). To improve vaccine safety, it is recommended to explore attenuated strains with well-characterized genotypes. Previously various genes have been mutated in SG toward developing a safe vaccine candidate including *aroA* (Griffin and Barrow, 1993), SPI-2 (Jones et al., 1998), *nuoG* (Zhang-Barber et al., 1998), *aroA-serC* (Barrow et al., 2000), *metC* (Shah et al., 2007), Δ*lon*Δ*cpxR* (Matsuda et al., 2011a), fur (Łaniewski et al., 2014), Δ*cobS*Δ*cbiA* (Penha Filho et al., 2010; Penha Filho et al., 2016) among others. In the early 1950s, Bacon and his colleagues observed that mutation in gene involved in purine production results in reduced virulence (Bacon et al., 1950a,b). Another experiment was conducted to evaluate the impact of various purine auxotrophic mutations on the virulence of a Vi-positive strain of *Salmonella* dublin and two strains of *Salmonella typhimurium* in mice (McFarland and Stocker, 1987). To our knowledge, the generation and safety assessment of purine-deficient *Salmonella* Gallinarum have not been previously documented. This study investigates the impact of *purB* deletion in SG on virulence, lesion scoring, and bacterial persistence, offering insights into its potential role in pathogenicity modulation. Specifically, we describe the creation of a single isogenic, markerless knockout mutant, denoted as SG Δ*purB*, engineered using *λ*-Red recombination. This mutant strain, lacking *purB*, disrupts adenylosuccinate lyase function, impacting purine biosynthesis and resulting in slower growth. The observed reduction in virulence in certain auxotrophic strains is attributed to compromised growth, impeding evasion of host defenses in contexts where essential metabolites are deficient (McFarland and Stocker, 1987). Further investigations are necessary to evaluate its immunogenicity and protective efficacy. Strategies like co-administration with adjuvants or combination with additional attenuating mutations may enhance its immunogenicity and effectiveness against *Salmonella* Gallinarum infection, addressing poultry health concerns.

## 2 Materials and methods

### 2.1 Bacterial strains and plasmids

Bacterial strains and plasmids employed in this study are delineated in Table 1. Local isolates of *Salmonella enterica* subsp. enterica serovar Gallinarum biovar Gallinarum (SG) (Accession no. CP150644) were sourced from the University Diagnostic Laboratory (UDL) of UVAS, Lahore. Plasmids utilized in the study, denoted as pCLF3 (EU629213), pKD46 and pCP20 (Datsenko and Wanner, 2000) were acquired from the McClelland laboratory, UCI, United States.

**Table 1:** Bacterial strains and plasmids used in study.

Bacterial isolate	Characteristics	References
*Salmonella* Gallinarum	*Salmonella enterica* subsp. enterica serovar Gallinarum biovar Gallinarum, isolated from an outbreak in the district Sheikhupura, Province Punjab, Pakistan	University Diagnostic laboratory, UVAS, Lahore.

Plasmid	Characteristics	References
pCLF3	Derivative of pKD3, Chloramphenicol resistance cassette, the PT7 promoter, and the FRT sites	Santiviago et al. (2009)
MG1655/pKD46	λ Red recombinase expression plasmid, repA101(ts), araBp-gam-bet-exo, bla (AmpR)	Department of Microbiology and Molecular Genetics, UCI, United States
EC100Δpir116/pCP20	AmpR, CmR, FLP recombinase expression	Department of Microbiology and Molecular Genetics, UCI, United States

### 2.2 Construction of single mutant Δ*purB* of *Salmonella* Gallinarum

The markerless isogenic single mutant Δ*purB*:ΩCm of SG was constructed by *λ*-Red-mediated recombination system (Datsenko and Wanner, 2000). The primer sequences employed for the generation and verification of mutant are detailed in Table 2.

**Table 2:** Primers used in study.

Gene name	Primer nature	Primer name	Primer sequence 5′---3′	Reference
*purB* (Red Primers)	Forward	*purB*-F	5′TGACCGCCGTTTCCCCTGTCGATGGACGCTACGGCGATAAAGTCAGCGCGGTGCAGGCTGGAGCTGCTTC3′	This study
Reverse	*purB*-R	5′ACCAGAGTCACAGCGCGACCGATATAATTTGCCGGCGTCATGGCTTTAAGCATATGAATATCCTCCTTAG3′	This study
*purB* flank	Forward	*purB* flank-F	5’TCATTTAACCCCGGAGTTAT3`	This study
Reverse	*purB* flank-R	5’TGAAGAAAAGAGGGTGAGGC3`	This study
Cassette-R			5’CTTCGAAGCAGCTCCAGCCTGCAC 3`	This study

#### 2.2.1 Amplification of *purB*-F_50_: Cm^R^: *purB*-R_50_

For targeted deletion of the *purB*, specific 70-base pair primers *purB*-F and *purB*-R were utilized to amplify segments containing the Chloramphenicol resistance marker along with Promoter T7 and FLP recombination target (FRT) sites from pCLF3. The resulting PCR products, flanked by 50-base homologous sequences at their ends, were designed to match regions proximal to the 5′ and 3′ ends of the *purB* gene. Cycling parameters included initial denaturation at 98°C for 1 min 30 s, followed by denaturation at 98°C for 15 s, annealing at 54°C for 20 s, and elongation at 72°C for 1 min 10 s for five cycles. Subsequently, there were 30 cycles of denaturation at 98°C for 15 s and elongation at 72°C for 1 min 30 s, with a final elongation step at 72°C for 3 min. After confirmation via gel electrophoresis, the amplified selection cassette was purified from the PCR mixture using the QIAquick PCR purification kit (QIAGEN, Germany).

#### 2.2.2 Electro-transformation of *Salmonella* Gallinarum with pKD46 (*λ* red plasmid)

The Lambda red plasmid pKD46 was extracted from MG1655 *Escherichia coli* using the commercially available plasmid extraction kit QIAprep^®^ Spin Miniprep Kit (QIAGEN, United States). *S. gallinarum* was prepared for electroporation using the subsequent protocol with some modifications (Cox et al., 2007) Datsenko and Wanner. The prepared electrocompetent cells were electroporated with the pKD46 plasmid at 1.8 kV in 1 mm cuvette (Bio-Rad, United States) for 5.7 milliseconds (ms) using EC1 on MicroPulser electroporator (Bio-Rad, United States), followed by recovery in SOC media and plating on LB medium supplemented with ampicillin (100 μg/mL). All resulting *Salmonella* Gallinarum transformants carrying the pKD46 plasmid were stored at-80°C for further analysis.

#### 2.2.3 Electro-transformation of *purB*-F_50_: Cm^R^: *purB*-R_50_ into SG: pKD46

SG cells harboring the pKD46 plasmid (46 μL) were inoculated into 23 mL of LB/Amp broth and incubated at 30°C with agitation for 1 h, followed by induction with 0.02% L-arabinose. Cells were grown to an OD_600_ of 0.47–0.48, and electrocompetent cells were prepared as described. Purified PCR product *purB*-F_50_: Cm^R^: *purB*-R_50_ having DNA concentration 1.5 μg (Serra-Moreno et al., 2006) was electroporated into electrocompetent cells of SG:pKD46 at 1.8 kV in a 1 mm cuvette (Bio-Rad, United States), followed by recovery in SOC media for 50 min at 37°C with shaking. Both, the cells harboring the *purB* (Cm^R^) marker and the cells subjected to the water control (IDT Nuclease-free water), were spread onto LB/Cm (15 μg/mL) plates and incubated overnight at 37°C (Karlinsey, 2007). Confirmation of SG Δ*purB*:ΩCm colonies was achieved through PCR amplification using *purB* flank-F, *purB* flank-R, and Cassette-R primers.

#### 2.2.4 Excision of resistance marker and curing of pCP20

To generate an isogenic markerless mutant, the Cm resistance marker was deleted following specific modifications (Czarniak and Hensel, 2015). A volume of 50 μL of competent cells of SG Δ*purB*:ΩCm was mixed with 3 μL of plasmid pCP20 in a pre-chilled electroporation cuvette and electroporated at 1.8 kV, followed by recovery in SOC media for 50 min at 30°C with shaking. Cells harboring the pCP20 plasmid were then spread onto LB/Amp (100 μg/mL) plates and incubated at room temperature. After 48 h, the cells were scraped, suspended in LB/20% glycerol, and diluted for overnight culture at 43°C. The culture was then streaked and spread on LB plates and incubated at 37°C. Eight colonies were selected and tested on LB/Cm (15 μg/mL) and LB/Amp (100 μg/mL) plates. The resulting SG Δ*purB* mutants were further verified by PCR and stored in 20% glycerol at-80°C.

### 2.3 Phenotypic characteristics of SG Δ*purB*

For the auxotrophic experiment, a single colony of SG Δ*purB* was cultured in 3 mL LB broth overnight at 37°C. Subsequently, 200ul of the overnight culture was inoculated into 20 mL of M9 minimal media, with one set of tubes supplemented with 0.3 mM adenosine and the other set without adenosine, incubated overnight at 37°C (Park et al., 2007; Jelsbak et al., 2014). To assess the *in vitro* growth pattern, a confirmed colony of SG Δ*purB* was inoculated into 3 mL LB broth and incubated overnight at 37°C. The following day, 200 μL of the overnight culture was transferred into 20 mL of LB broth, and optical density (OD_600_) was measured every hour for a duration of 10 h. The growth pattern of the wild-type SG was similarly observed as a control under the same conditions (Kang et al., 2022).

### 2.4 Animal ethics and husbandry conditions

The chicken experiments in this study were approved by the Animal Ethics Committee of UVAS and conducted in accordance with institutional ethical guidelines. Disease-free broiler chickens were obtained as day-old chicks from a commercial hatchery and housed in pre-sterilized pens within an environmentally controlled room, provided with water and antibiotic-free food *ad lib.*

### 2.5 Assessment of bacterial virulence

This experiment involved the evaluation of SG wild-type strain and SG Δ*purB* virulence by administering different doses of each strain orally to chickens. One hundred and ten day-old chickens were divided into three major groups (Group A, B, and C). Groups A and B, each comprising fifty chickens, were further subdivided into subsequent sub-groups, each containing ten birds. Group C consisted of 10 birds. On day 3, each sub-group of Group A (*n* = 10) received a 10-fold dilution (ranging from 1 × 10^5^ to 1 × 10^9^) dose of SG Δ*purB*, while each sub-group of Group B was inoculated with a 10-fold dilution (1 × 10^5^ to 1 × 10^9^) dose of SG wild-type via oral route in 100 μL PBS. Ten birds in group C were inoculated with 100 μL PBS as a control group. Bird mortality was monitored for duration of 2 weeks. The Lethal dose (LD_50_) was calculated by Probit analysis (Finney, 1971).

#### 2.5.1 General condition, mortality and gross lesion observations

In this experiment, ninety *Salmonella*-negative day-old chickens were allocated into three groups. On day 3, Group A and Group B were orally inoculated with the SG Δ*purB* mutant and SG wild-type strains, respectively, at a dosage of 2 × 10^8^ CFU per bird in 100 μL PBS, while Control Group C received 100 μL PBS via the same route. Body weights of the birds were recorded at 0, 7, 14, 21, and 28 days post-infection (DPI) (Kang et al., 2022). Throughout the 28-day observation period, clinical symptoms and lesion scores were monitored. Gross lesions in the spleen and liver were assessed through post-mortem examination of 5 randomly selected birds from each group on 7 DPI, with the remaining chickens being humanely slaughtered at the trial’s conclusion. Clinical symptoms, such as depression and diarrhea, were monitored daily from 5DPI to 10DPI. The scoring criteria for both clinical symptoms and gross lesions were based on methods described in a previous study (Matsuda et al., 2011b).

#### 2.5.2 Persistence of bacteria in liver and spleen

Bacteriological examination of the organs was conducted to assess bacterial persistence. Three birds from each group were slaughtered at 3, 7, 10, 14 and 21 DPI, and samples of the liver and spleen were collected aseptically in sterile zip bags. One gram samples of the liver and spleen were minced and homogenized in 1 mL of PBS. Subsequently, 10-fold serially diluted samples (100 μL) were inoculated onto BGA and XLD agar plates, which were then incubated at 37°C for 24 h. Additionally, homogenized samples in PBS were inoculated into Rappaport-Vassiliadis broth at 42°C for 24 to 48 h for enrichment, followed by inoculation of 100 μL onto BGA agar plates, which were incubated at 37°C for 24 h. Confirmation of the mutant strain (SG Δ*purB*) and SG wild-type from the samples was achieved via PCR using specific primers *purB* flank-F/*purB* flank-R. The persistence of SG Δ*purB* and SG wild-type was quantified and expressed in log_10_ CFU/g. Samples that tested positive only after enrichment were considered as 1 CFU/g, while those that remained negative after enrichment were considered as 0 CFU/g for data analysis (Matsuda et al., 2010).

## 3 Results

### 3.1 Molecular validation of *purB* deletion in *Salmonella* Gallinarum

The amplification of target sequences with *purB*-F and *purB*-R primers resulted in 1180 bp PCR product, designated as *purB*-F_50_:Cm^R^:*purB*-R_50_. This purified PCR product (1.5 μg) when transformed into SG:pKD46, produced 15 colonies on LB/Cm (15 μg/mL) plates, whereas, no colonies were observed in the water control. PCR screening of SG Δ*purB*:ΩCm mutants demonstrated a 1,222 bp product in the mutant strain and a 1,421 bp product in the wild-type strain using *purB* flank-F/*purB* flank-R primers, confirming the inactivation of *purB* (Supplementary Figure S1). Additionally, amplification with *purB* flank-F/Cassette-R primers generated a 114 bp product in the mutant strain, verifying the insertion of the chloramphenicol resistance cassette at the target site (Supplementary Figure S2). The antibiotic cassette inserted into the inactivated *purB* gene was removed using FLP recombinase, resulting in the intended 298 bp genomic scars, as confirmed by PCR using specific flanking primers *purB* flank-F/*purB* flank-R (Supplementary Figure S3).

### 3.2 Auxotrophic evaluation and growth dynamics of SG Δ*purB*

**Figure 1:**
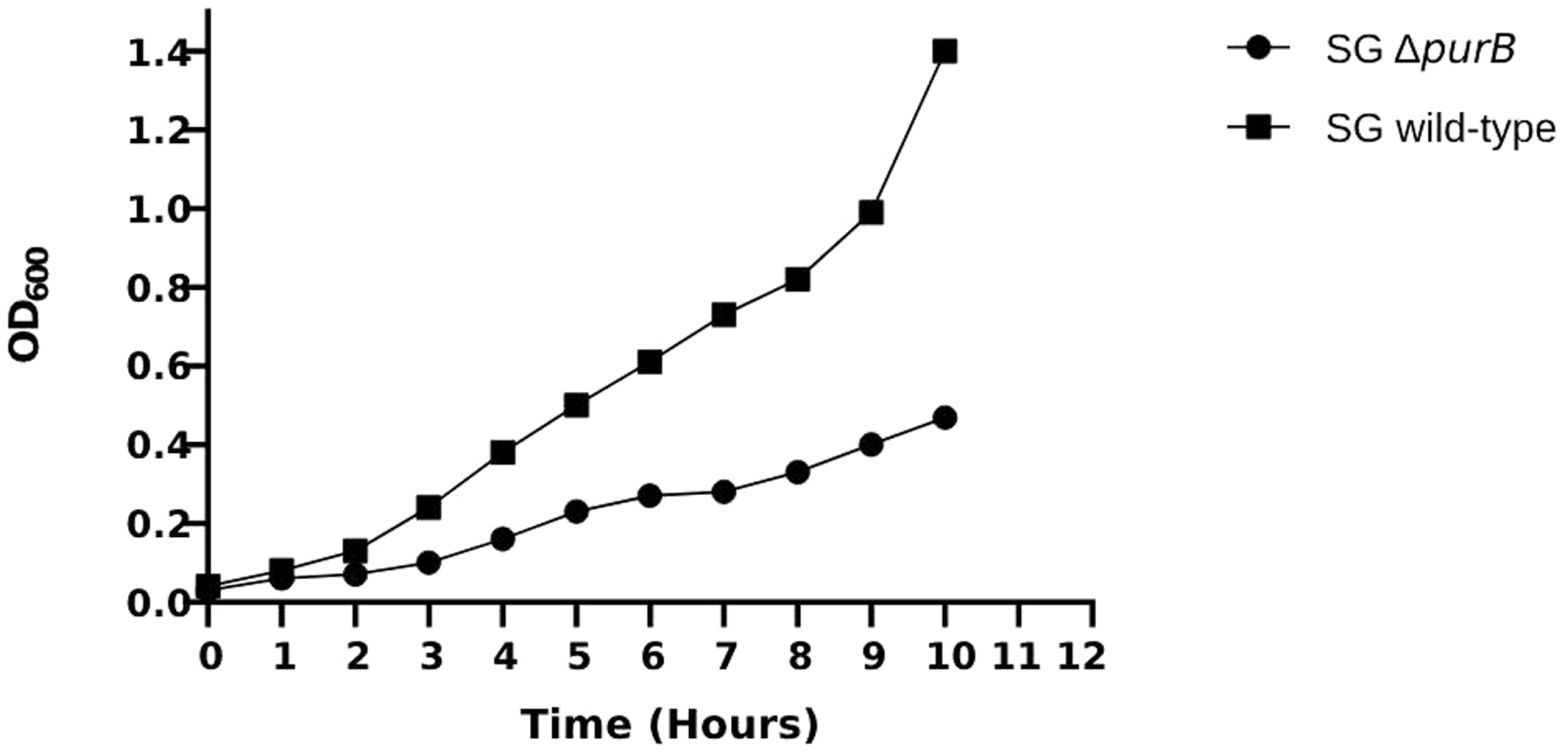
Growth patterns of the SG Δ*purB* mutant and the SG wild-type strains in LB broth over a 10 h period. A significant difference in growth between the SG Δ*purB* mutant and the SG wild-type was observed at the 10 h mark, as determined by a two-tailed Student’s *t*-test with equal variance (*p* = 0.02).

**Figure 2:**
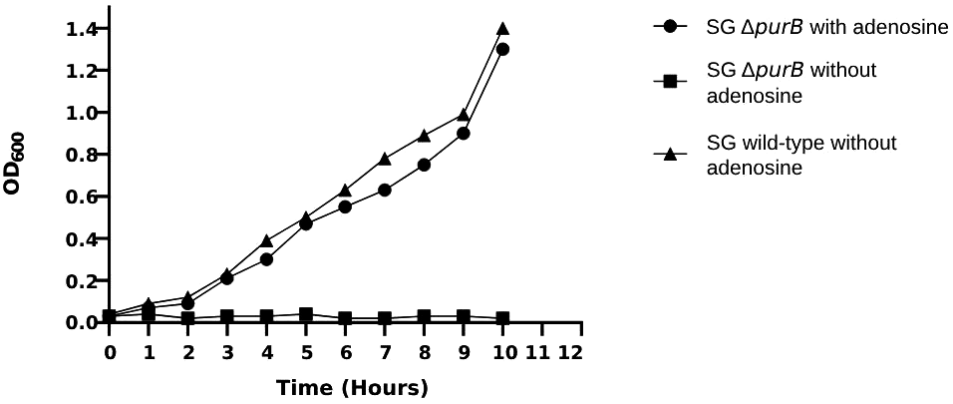
Growth dynamics of the SG Δ*purB* mutant and SG wild-type strains in M9 minimal media, with and without adenosine supplementation. The growth curve of SG Δ*purB* supplemented with adenosine showed no significant difference compared to SG wild-type without adenosine (*p* = 0.7). However, a significant difference was observed between the growth curve of SG Δ*purB* and SG wild-type without adenosine supplementation (*p* < 0.05), as determined by a *t*-test.

The growth dynamics of SG Δ*purB* mutant and SG wild-type were monitored over a 10 h period in LB broth. Following the specified duration, the optical density at 600 nm (OD_600_) for SG Δ*purB* reached 0.47, reflecting a notable 66.5% reduction compared to the robust OD_600_ of 1.40 observed for the SG wild-type (*p* = 0.02; Figure 1). The growth dynamics of the SG Δ*purB* mutant in M9 minimal media, with or without supplemented adenosine, were evaluated. At the time of inoculation, the SG Δ*purB* mutant exhibited an initial OD_600_ of 0.03. After ten hours of incubation, the SG Δ*purB* mutant showed no significant growth, maintaining an OD_600_ of 0.02. In contrast, the SG wild-type reached an OD_600_ of 1.40 under the same conditions (*p* = 0.7). Notably, when the M9 minimal media was supplemented with 0.3 mM adenosine, the OD_600_ of the SG Δ*purB* mutant increased significantly to 1.30, closely approaching the OD_600_ of 1.40 observed for the SG wild-type (Figure 2).

### 3.3 Virulence assessment of SG Δ*purB* mutant vs. SG wild-type

Virulence was evaluated in 3-day old chickens by calculating LD_50_ of SG Δ*purB* and SG wild-type. LD_50_ of SG wild-type was 2 × 10^8^ CFU/mL whereas no chickens died in the group challenged by SG Δ*purB.*

**Figure 3:**
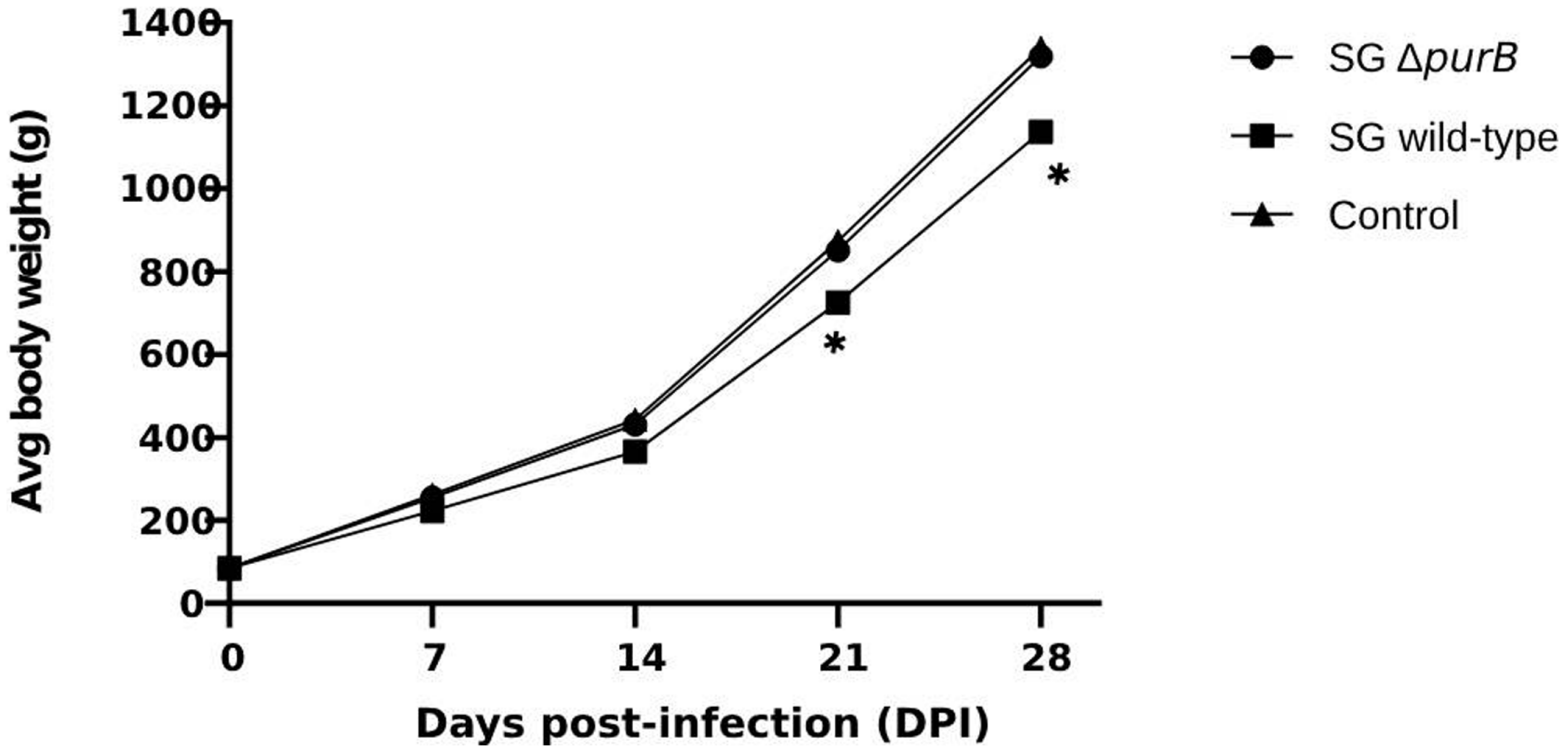
Body weight changes of chickens following infection with SG Δ*purB*, SG wild-type, and a control group administered 100 μL of PBS. Body weights were measured at 0, 7, 14, 21, and 28 days post-infection (DPI). A significant difference was observed in the group infected with SG wild-type on 21 and 28 DPI compared to the group infected with SG Δ*purB* and the uninfected control group (* *p* < 0.05), as determined by two-way ANOVA followed by Bonferroni’s multiple comparison tests. Data are presented as mean ± SEM.

**Figure 4:**
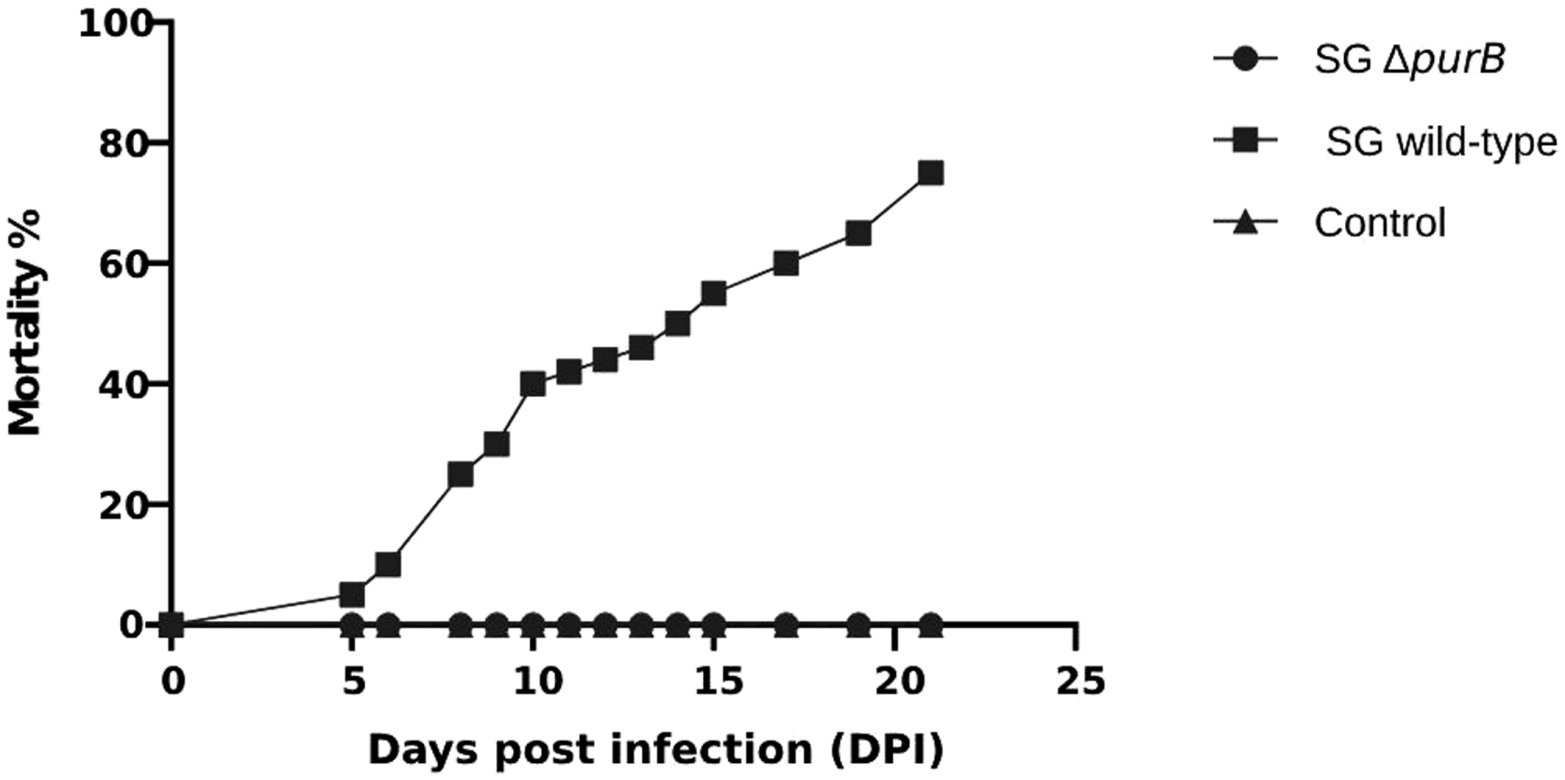
Mortality of chickens observed after infection with SGΔ*purB*, SG wild-type, and control groups administered PBS during the experimental period. The group infected with SG wild-type exhibited 80% mortality, whereas no mortality was observed in the group infected with SGΔ*purB* and the uninfected control group.

Following the oral administration of 2 × 10^8^ CFU in 100 μL PBS of SG Δ*purB* and SG wild-type to 3-day old chickens, body weight changes were monitored weekly, with values expressed as Mean ± SEM and analyzed using GraphPad Prism 10 software. The data, presented in Table 3, showed that Group B, inoculated with SG wild-type, demonstrated significantly lower body weights than control of 84.4 ± 0.53 g, 221.9 ± 0.60 g, 365.9 ± 0.84 g, 724.5 ± 0.70 g, and 1137.3 ± 0.57 g at 0, 7, 14, 21, and 28 DPI, respectively (*p* = 0.01). Group A, inoculated with SG Δ*purB*, exhibited body weights of 84.6 ± 0.30 g, 255.1 ± 0.80 g, 430.5 ± 0.84 g, 851.2 ± 0.72 g, and 1318.6 ± 1.19 g at 0, 7, 14, 21, and 28 DPI, respectively. These values were similar to those of the control group (Group C), which received 100 μL PBS and showed weights of 84.3 ± 0.36 g, 262 ± 0.89 g, 442.5 ± 0.45 g, 873.5 ± 0.833 g, and 1,340 ± 1.11 g at the same intervals (*p* = 0.7), which indicate that SG Δ*purB* has significantly reduced its ability to cause detrimental effect on chicken growth (Figure 3). Mortality was also recorded throughout the study period, as illustrated in Figure 4. Both Group C (PBS control) and Group A (SG Δ*purB*) exhibited no mortality, whereas Group B (SG wild-type) experienced a high mortality rate of 80%. These findings underscore the marked attenuation of virulence in the SG Δ*purB* mutant relative to the wild-type strain, as well as its non-lethal effects on the growth performance of the host.

**Table 3:** Comparison of body weight changes in chickens following infection with SG Δ*purB* and SG wild-type.

Groups	*n*	Body weight changes (g)				
		0DPI	7DPI	14DPI	21DPI	28DPI
SG Δ*purB*	20	84.6 ± 0.30	255.1 ± 0.80	430.5 ± 0.84	851.2 ± 0.72	1318.6 ± 1.19
SG wild-type	20	84.4 ± 0.53	221.9 ± 0.60	365.9 ± 0.84	724.5 ± 0.70	1137.3 ± 0.57
PBS	20	84.3 ± 0.36	262 ± 0.89	442.5 ± 0.45	873.5 ± 0.83	1,340 ± 1.11

### 3.4 Clinical manifestations and macroscopic lesions

**Figure 5:**
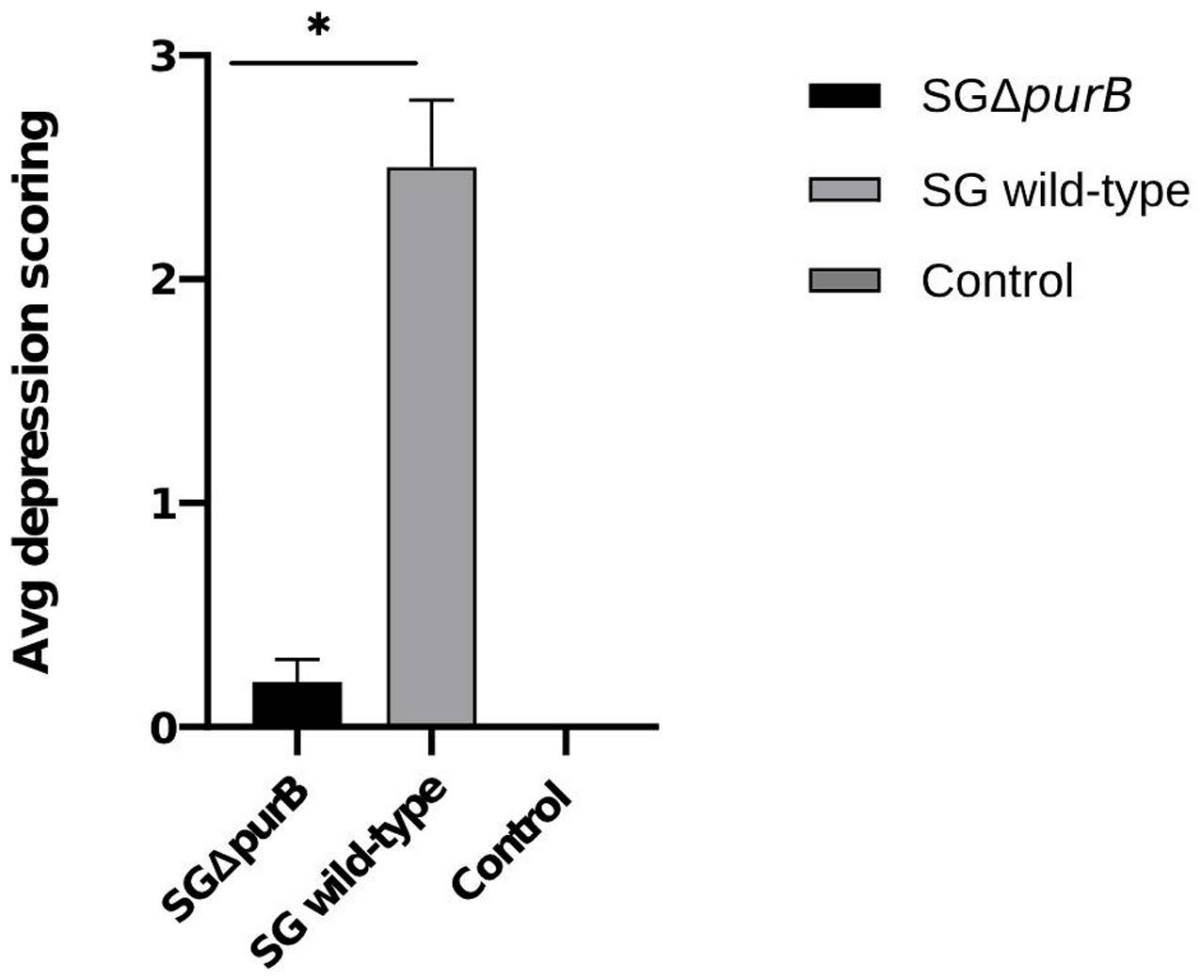
Maximum depression scores of all groups from 5 DPI to 10 DPI, represented as Mean ± SEM. The group infected with SG wild-type (grey bar) showed significantly severe depression scores from 5 DPI compared to the group infected with SG Δ*purB* (black bar) (Mann–Whitney U-test, * *p* < 0.05). The uninfected control group showed no clinical signs.

Clinical observations were conducted bi-daily across all experimental groups (Groups A, B, and C) as delineated in Table 4, with findings presented as mean ± SEM. Notably, chickens in Group B (inoculated with SG wild-type) exhibited significantly heightened depression scores from 5 DPI to 10 DPI compared to Group A, as determined by the Mann- Whitney U-test (*p* = 0.001). The peak depression scores recorded during the study period for Groups A, B, and C were 0.2 ± 0.1, 2.5 ± 0.3, and 0.0 ± 0.0, respectively (Figure 5).

**Table 4:** Clinical sign scoring after infection.

Groups	Depression scoring	Diarrheal scoring
SG *ΔpurB* (A)	0.2 ± 0.1	0.4 ± 0.2
SG wild-type (B)	2.5 ± 0.3	2.7 ± 0.2
Control (C)	0.0 ± 0.0	0.0 ± 0.0

**Figure 6:**
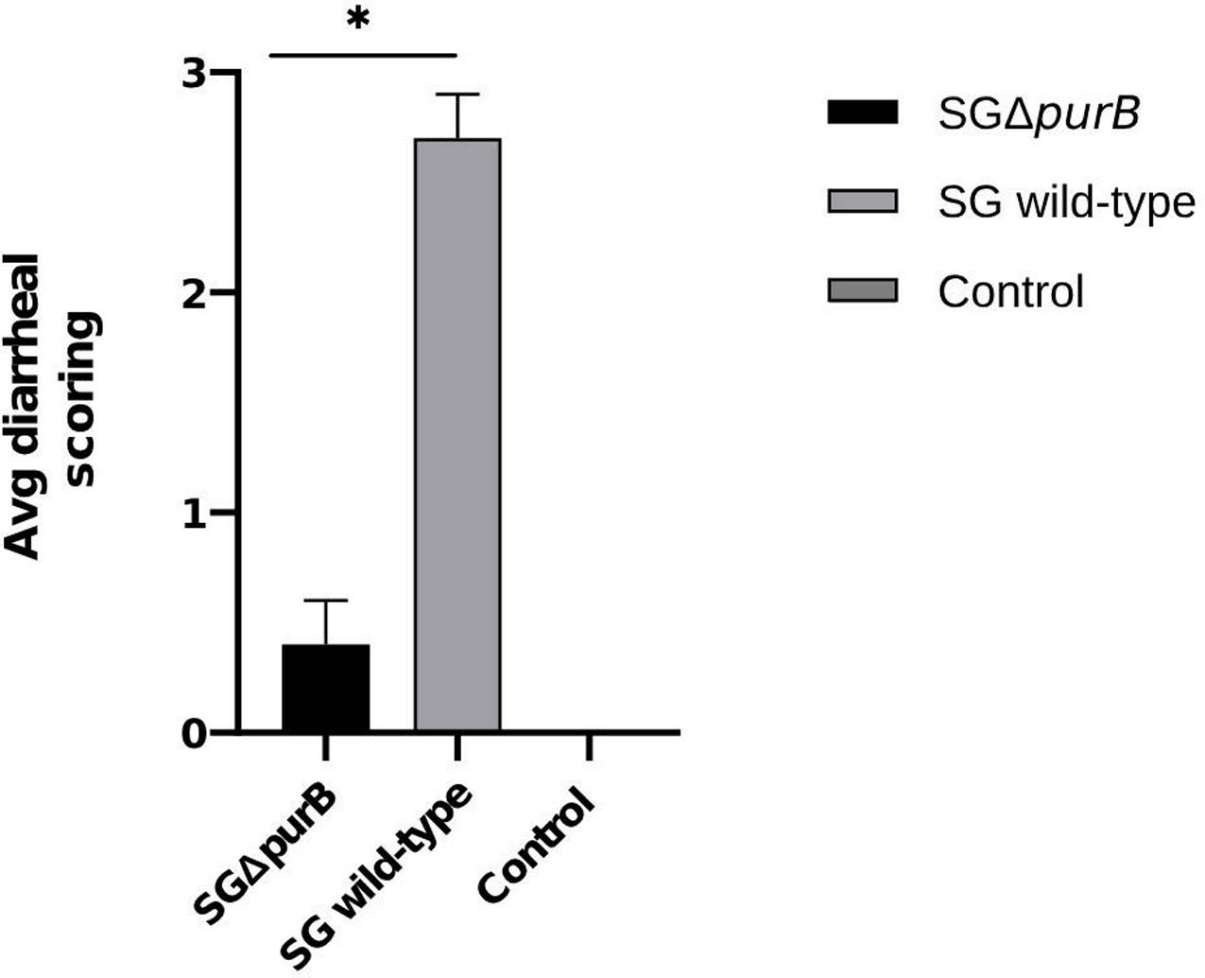
Maximum diarrheal scores of all groups from 5 DPI to 10 DPI, represented as Mean ± SEM. The group infected with SG wild-type (grey bar) showed significantly severe diarrheal scores compared to the group infected with SG Δ*purB* (black bar) (Mann–Whitney U-test, * *p* < 0.05). The uninfected control group showed no clinical signs.

**Figure 7:**
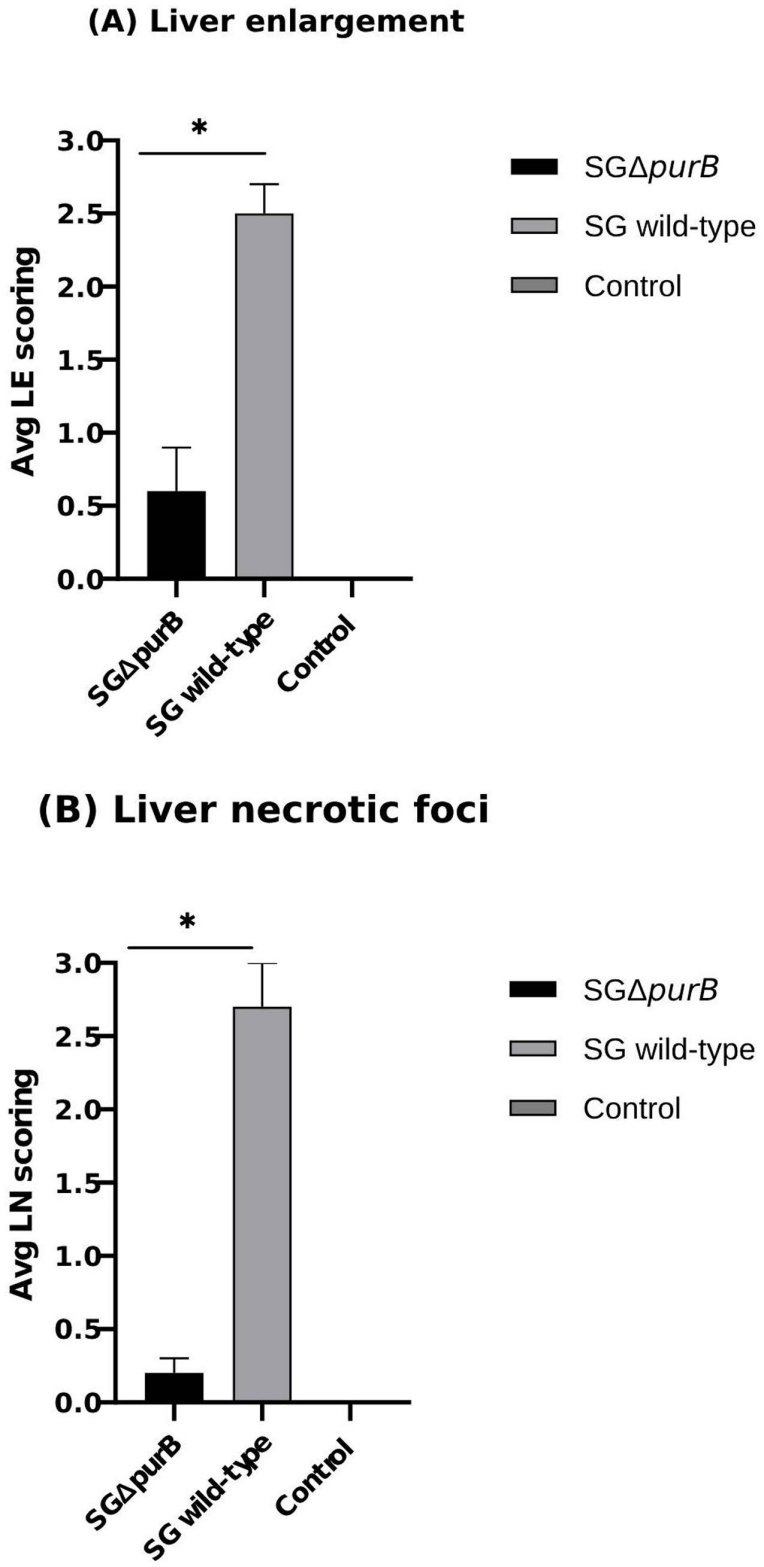
Gross liver lesions were observed at 7 DPI. **(A)** Mean liver enlargement scores in the SG wild-type group (grey bar) were significantly higher than in the SG Δ*purB* group (black bar), as measured by the Mann–Whitney U-test, * *p* < 0.05 (LE scoring: 0- Clear ridge; 1- Soft tissue; 2- Large without covering gizzard; 3- Large covering gizzard). **(B)** Mean liver necrotic foci scores in the SG wild-type group (grey bar) were significantly higher than in the SG Δ*purB* group (black bar), as measured by the Mann–Whitney U-test, * *p* < 0.05 (LN scoring: 0- No foci; 1- < 5 foci; 2- < 20 foci; 3- > 20 foci). Data are presented as mean ± SEM. The uninfected control group showed no gross lesions.

**Figure 8:**
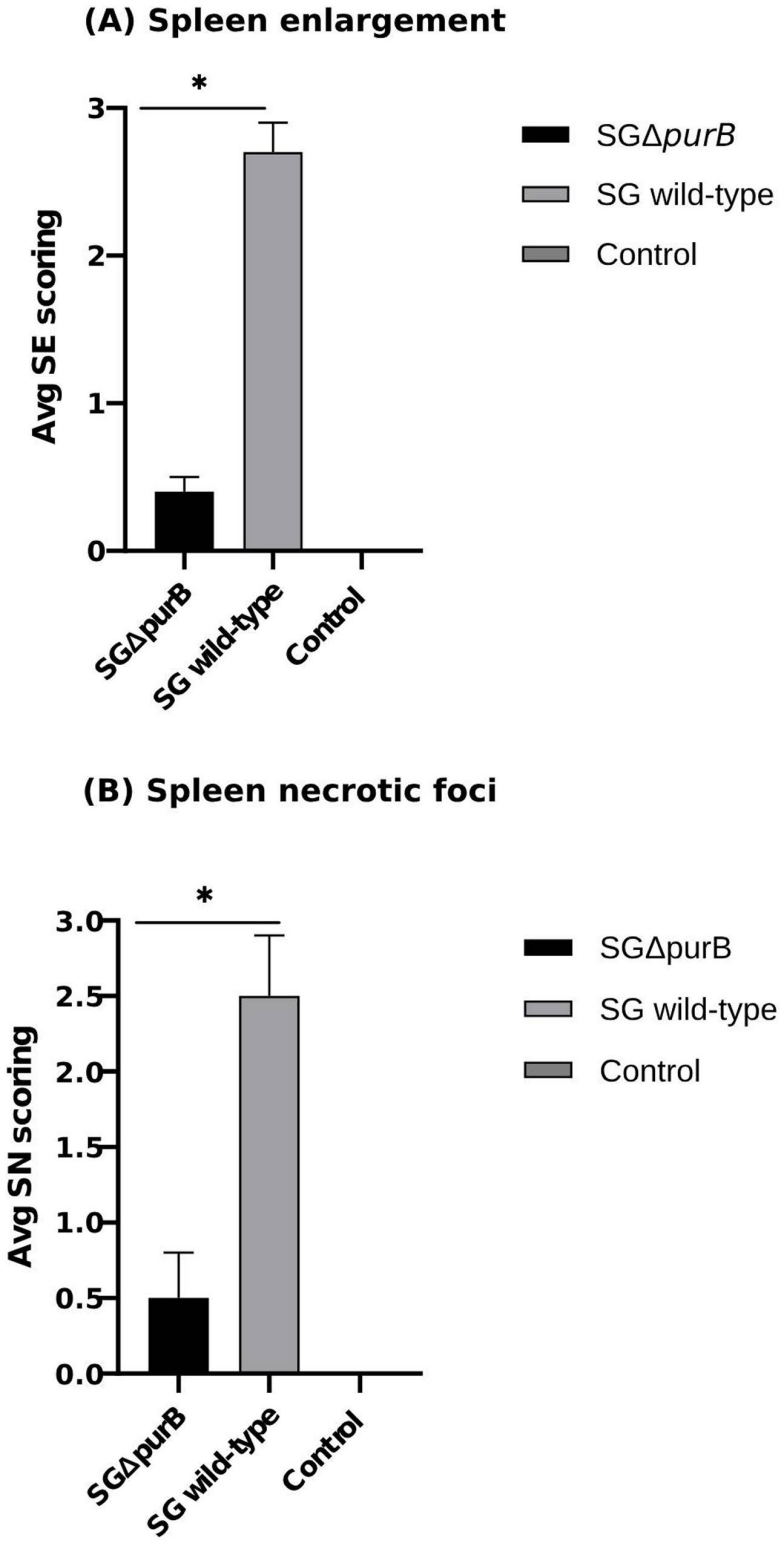
Gross lesions on the spleen were observed on 7 DPI. **(A)** Mean spleen enlargement scores in SG wild-type group (grey bar) was significantly higher than SG Δ*purB* group (black bar) as measured by Mann–Whitney U-test, * *p* < 0.05 (SE scoring, 0-not soft; 1-soft without enlargement; 2-large with <1.5 dm; 3-large with >1.5 dm) **(B)** Mean spleen necrotic foci scores in SG wild-type group (grey bar) was significantly higher than SG Δ*purB* group (black bar) as measured by Mann–Whitney U-test, * *p* < 0.05 (SN scoring, 0- No foci; 1- if <3 foci; 2- if <10 foci; 3- if >10 foci), data presented as (Mean ± SEM). The uninfected control group had no gross lesions.

Similarly, Group B also demonstrated significantly more severe diarrheal symptoms compared to Group A, as evidenced by Mann–Whitney U-test results (*p* = 0.001). The maximum diarrheal scores observed for Groups A, B, and C throughout the experiment was 0.4 ± 0.2, 2.7 ± 0.2, and 0.0 ± 0.0, respectively, as depicted in Figure 6. To observe the gross lesions, spleen and liver was collected from 5 randomly chickens each group on the 7 DPI. Group B showed significantly severe systemic infection when compared with Group A according to Mann–Whitney U-test (*p* = 0.001) and data presented as Mean ± SEM as shown in Table 5. Mean score for liver enlargement in group A (SG Δ*purB*) and group B (SG wild-type) was 0.6 ± 0.3 and 2.5 ± 0.2, respectively. Whereas mean score for liver necrotic foci in the group A and group B was 0.2 ± 0.1 and 2.7 ± 0.3, respectively. Similarly, the mean score for spleen enlargement in the group A and group B was 0.4 ± 0.1 and 2.7 ± 0.4, respectively. Whereas the mean score for spleen necrotic foci in group A and group B was 0.5 ± 0.3 and 2.5 ± 0.4, respectively. Group C inoculated with PBS, a control group was negative for all gross lesions as shown in Table 5. Graphical presentation also showed in Figures 7, 8.

**Table 5:** Gross lesions in liver and spleen after infection.

Groups	*n*	Gross lesions after infection			
		LE	LN	SE	SN
SG *ΔpurB*	5	0.6 ± 0.3	0.2 ± 0.1	0.4 ± 0.1	0.5 ± 0.3
SG wild-type	5	2.5 ± 0.2	2.7 ± 0.3	2.7 ± 0.4	2.5 ± 0.4
Control	5	0.0 ± 0.0	0.0 ± 0.0	0.0 ± 0.0	0.0 ± 0.0

### 3.5 Duration of SG retention in liver and spleen

**Figure 9:**
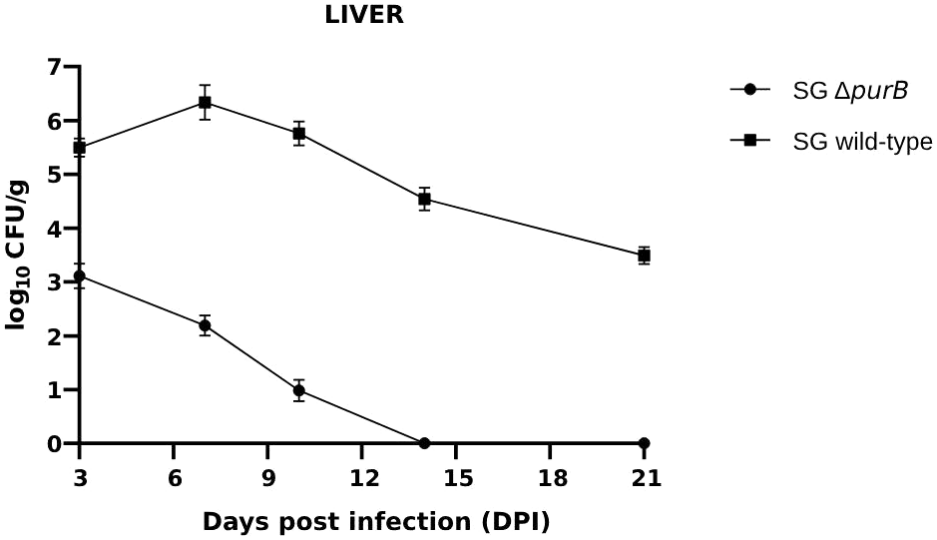
Bacterial colonization in the liver was assessed at 3, 7, 10, 14, and 21 days post-infection (DPI). Bacterial counts are expressed as the log_10_ of CFU/g. Each data point represents the mean of five samples, with data presented as mean ± SEM. Bacterial persistence in the group infected with the SG wild-type strain was significantly higher than in the group infected with the SG Δ*purB* strain, as determined by two-tailed *t*-tests *p* < 0.05. The SG Δ*purB* strain was cleared from the liver by 14 DPI.

**Figure 10:**
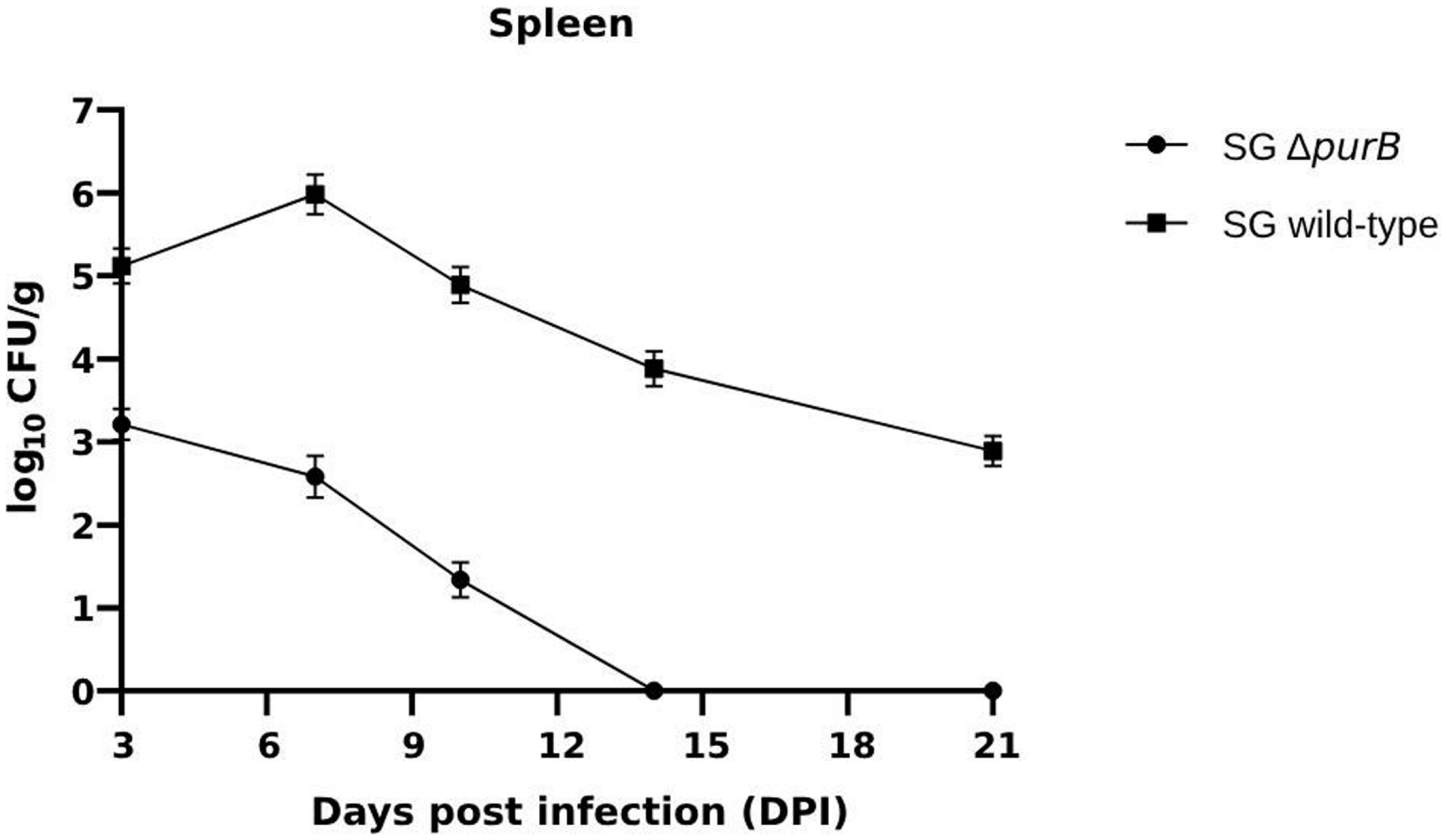
Bacterial colonization in the spleen was assessed at 3, 7, 10, 14, and 21 days post-infection (DPI). Bacterial counts are expressed as the log10 of CFU/g. Each data point represents the mean of five samples, with data presented as mean ± SEM. Bacterial persistence in the group infected with the SG wild-type strain was significantly higher than in the group infected with the SG Δ*purB* strain, as determined by two-tailed *t*-tests *p* < 0.05. The SG Δ*purB* strain was cleared from the spleen by 14 DPI.

Bacterial colonization and persistence within the liver and spleen were systematically evaluated at 3, 7, 10, 14 and 21 days post-infection (DPI). The control group showed no *Salmonella* recovery from either organ following enrichment in Rappaport Vassiliadis broth, confirming the specificity of the infection model. In contrast, colonization by SG Δ*purB* was markedly reduced in both the liver and spleen throughout the study period, demonstrating significantly lower bacterial counts (*p* < 0.05) compared to the SG wild-type. Quantitative analysis revealed that in Group A (SG Δ*purB*), the bacterial load in the liver measured 3.11 ± 0.23, 2.19 ± 0.19, 0.98 ± 0.20, 0.0 ± 0.0, and 0.0 ± 0.0 CFU/g, and in the spleen 3.21 ± 0.19, 2.58 ± 0.25, 1.34 ± 0.21, 0.0 ± 0.0, and 0.0 ± 0.0 CFU/g at 3, 7, 10, 14, and 21 DPI, respectively (Figure 9; Table 6). Conversely, Group B (SG wild-type) exhibited consistently higher CFU/g in the liver (5.50 ± 0.17, 6.34 ± 0.32, 5.76 ± 0.22, 4.54 ± 0.21, 3.49 ± 0.16) and spleen (5.12 ± 0.21, 5.98 ± 0.24, 4.89 ± 0.22, 3.88 ± 0.21, 2.89 ± 0.29) at corresponding time points (Figure 10; Table 6).

**Table 6:** Bacterial persistence in liver and spleen after infection.

Groups	*n*	DPI	Log_10_ mean counts per gram	
			Liver	Spleen
SG Δ*purB*	3	3	3.11 ± 0.23	3.21 ± 0.19
	3	7	2.19 ± 0.19	2.58 ± 0.25
	3	10	0.98 ± 0.20	1.34 ± 0.21
	3	14	0.0 ± 0.0	0.0 ± 0.0
	3	21	0.0 ± 0.0	0.0 ± 0.0
SG wild-type	3	3	5.50 ± 0.17	5.12 ± 0.21
	3	7	6.34 ± 0.32	5.98 ± 0.24
	3	10	5.76 ± 0.22	4.89 ± 0.22
	3	14	4.54 ± 0.21	3.88 ± 0.21
	3	21	3.49 ± 0.16	2.89 ± 0.18

## 4 Discussion

Fowl Typhoid (FT), a septicemic disease induced by *Salmonella* Gallinarum (SG), causes substantial economic setbacks to the poultry industry worldwide (Matsuda et al., 2011b). Previous studies demonstrated that SG is transmitted both vertically and horizontally (Berchieri et al., 2001). To control and prevent *Salmonella* infections within poultry flocks, vaccination of chickens is a helpful method in addition to adequate management, good agricultural practices, and stringent biosecurity measures (Revolledo and Ferreira, 2012). When it comes to eliciting an immune response against *Salmonella* infection, live attenuated vaccines work better than inactivated or subunit vaccines (Barrow, 2007). In the current investigation, we endeavored to develop a single markerless isogenic mutant denoted as SG Δ*purB* and subsequently assess its safety, virulence, lesion scoring, and bacterial colonization as a potential live attenuated vaccine candidate. To the best of our knowledge, this is the first example of purine biosynthesis deficient mutant in SG and use of chicken as an infection model for *purB* mutant.

The current study demonstrated the attenuation of SG through the deletion of the *purB* gene, which is responsible for encoding adenylosuccinate lyase—a key enzyme in the final step of the purine biosynthesis pathway that converts adenylosuccinate to AMP. Previously, studies have been conducted on mutations in the purine biosynthesis pathway in the *purE* and *purH*, *purD* genes of *Brucella melitensis* and *Shigella flexneri*, respectively. These auxotrophic mutants were unable to grow in minimal media without purine supplements (Drazek et al., 1995; Crawford et al., 1996; Cersini et al., 2003). It was also demonstrated that auxotrophic mutants exhibit slow growth due to insufficient nutrient availability (McFarland and Stocker, 1987). In the present study, deletion of *purB* in SG resulted in 66.5% reduction in growth in LB broth when compared to wild-type. In addition, SG Δ*purB* could not maintain its growth in minimal media but restored its growth when supplemented with 0.3 mM adenosine in minimal media. This phenotypic characteristic confirms that *purB* mutant growth was impaired due to inability of the mutant to produce its endogenous adenosine and requires exogenous adenosine for normal growth which might be due to deficiency of purine biosynthesis as studied previously (Park et al., 2007).

Previous studies in a mouse model showed that purine biosynthesis is crucial for *Listeria monocytogenes* virulence, as *purB* mutants exhibited reduced virulence due to impaired multiplication within intestinal epithelial cells (Faith et al., 2012). To study virulence of SG in chicken model, a previously developed lesion scoring method was adopted (Matsuda et al., 2011b). The lesions in the liver and spleen, such as necrotic foci and hepatosplenomegaly caused by lymphocyte infiltration, are characteristic of SG infection. However, their role in protective immunity remains unclear, likely due to the acute pathogenicity of SG in chickens (Permin and Bisgaard, 2013). In this study, clinical signs and lesion scores were significantly higher in chickens infected with the SG wild-type compared to those infected with the SG Δ*purB* mutant, indicating significant attenuation of pathogenicity due to the mutation. Oral inoculation with SG Δ*purB* resulted in minimal adverse effects, with only a few small necrotic foci observed on the liver and spleen within a few days post-inoculation. Additionally, by 7 days post-inoculation (DPI), there was moderate enlargement of the spleen and liver in the SG Δ*purB*-infected group, with recovery observed by 14 DPI. These mild lesions are likely due to a cellular immune response rather than significant tissue and functional disturbances. The significant attenuation observed in the SG Δ*purB* mutant aligns with previous findings that mutations introducing new auxotrophic requirements can reduce virulence by hindering bacterial growth in host tissues (Lan et al., 2010). According to previous study (Christensen et al., 1996), the presence of gross lesions and clinical symptoms may be related to the quantity of completely virulent SG in the spleen. However, in cases when the host is resistant or the SG is attenuated, the spleen can remove the SG without causing severe clinical symptoms that would indicate the peak of the immune response (Wigley et al., 2005). To determine if the attenuation of virulence in the *purB* mutant was due to reduced invasiveness or a lower microbial burden in reticuloendothelial organs, we compared the *purB* mutant persistence in liver and spleen, with that of the wild-type strain. The *purB* mutant bacteria were present in the livers and spleens from day 3 post-inoculation, but their numbers were significantly lower (*p* < 0.01) than those of the WT strain. This indicates that the deletion of the *purB* gene reduced SG virulence by decreasing the bacterial burden in the liver and spleen of chickens. Importantly, the mutant bacteria were undetectable in the spleen and in the liver from day 14 onwards, suggesting a significant reduction in *in vivo* growth rate. This reduction is likely due to inadequate adenosine availability in the chicken intestinal tract and/or more effective elimination by the host’s immune defense mechanisms as mentioned in a previous study (Shah et al., 2007). Similarly, in our study, the body weights of chicks infected with SG Δ*purB* closely paralleled those of the control group, whereas the weights of the wild-type infected group were significantly lower at 21 and 28 days post-infection (DPI). This weight reduction in the wild-type infected group was associated with high fever, reduced feed intake as described in previous study (Audisio and Terzolo, 2002) and severe clinical signs starting from 5 days post-inoculation, underscoring that *purB* deletion effectively diminishes SG virulence.

Our study shows that SG Δ*purB* caused minimal adverse effects, with only a few minor necrotic foci in the liver and spleen, and are rapidly cleared from these organs within 14 DPI. Infected birds recovered swiftly, indicating a markedly attenuated phenotype which ensures its safety. Further studies would be required to determine the immunogenicity of this strain when used as a vaccine candidate. These findings underscore the promise of SG Δ*purB* as a foundation for developing optimized attenuated strains for vaccine design, against *Salmonella* Gallinarum, addressing key poultry health concerns.

## 5 Conclusion

Our investigation demonstrates a significant reduction in virulence of the *purB*-deleted SG strain in chickens following oral inoculation. This strain exhibited early organ clearance, reduced lesion severity, enhanced safety, and no adverse impact on chicken growth rate or weight gain, poses it as a promising vaccine candidate.

## Data Availability

The original contributions presented in the study are included in the article/Supplementary material, further inquiries can be directed to the corresponding author.
