# Supplementary material for: Construction, molecular characterization, and safety assessment of purB mutant of Salmonella Gallinarum
Source: Front Microbiol. 2024 Nov 13;15:1467230. doi: 10.3389/fmicb.2024.1467230 (PMC11599157; doi:10.3389/fmicb.2024.1467230)
Supplement: Supplementary file 1 [file Table_1.DOCX]

Supplementary Material

**.**

**M 1 2 3 4 5**

**
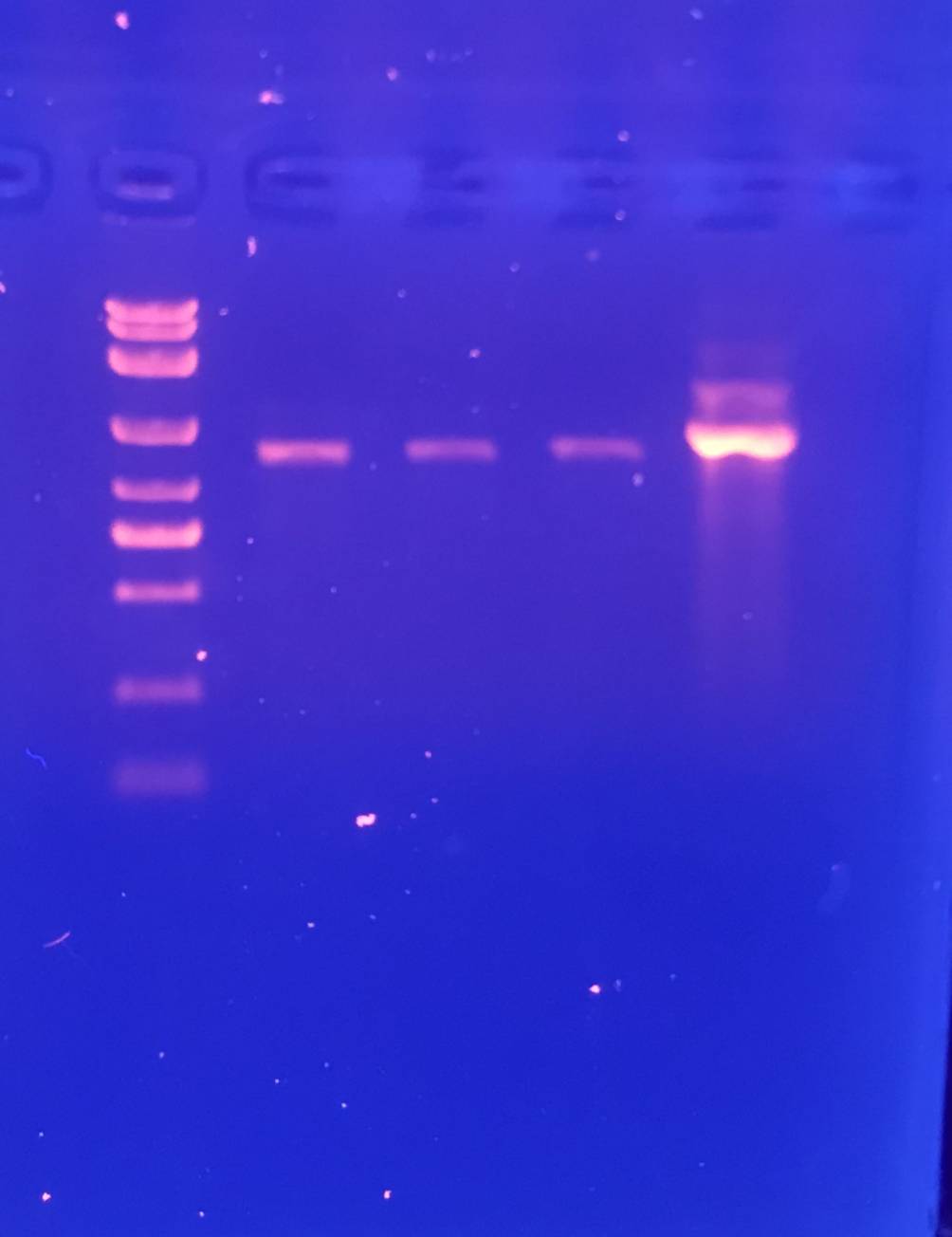
**

1500

1000

750

**1421 bp**

**1222 bp**

100

**Supplementary figure 1:** Gel electrophoresis analysis of PCR-amplified products demonstrating the replacement of the *purB* gene with the ΩCm cassette. Lane M: Lambda Biotech 1 Kb DNA ladder. Lanes 1-3: PCR products of SG Δ*purB*:ΩCm mutant showing insertion of the cassette (1222 bp). Lane 4: wild-type SG control (1421 bp). Lane 5: negative control (no template).

**M 1 2 3 4 5 6 7 8 9**


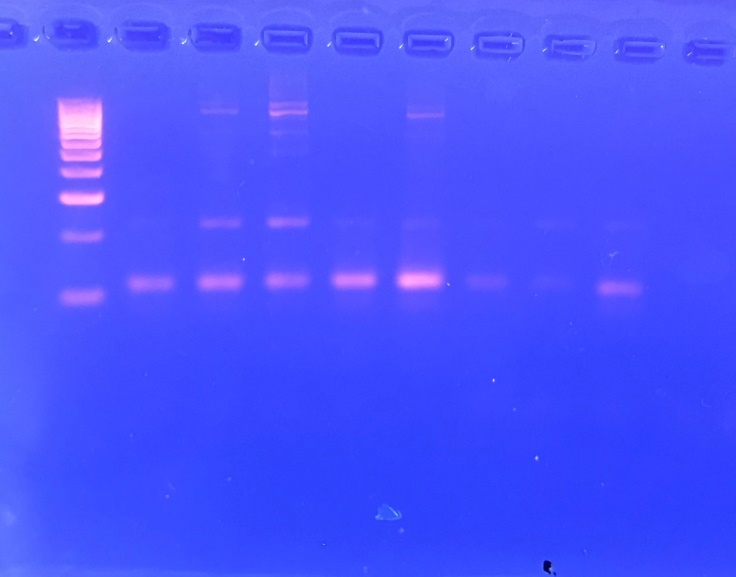


200

**114 bp**

100

**Supplementary Figure 2.** Gel electrophoresis analysis of PCR-amplified products demonstrating the replacement of the *purB* gene with the ΩCm cassette. Lane M: HyperLadder™ 100bp DNA ladder. Lanes 1-8: PCR products of SG Δ*purB*:ΩCm using *purB*/flank-F and Cassette-R primers (114 bp). Lane 9: negative control (no template).


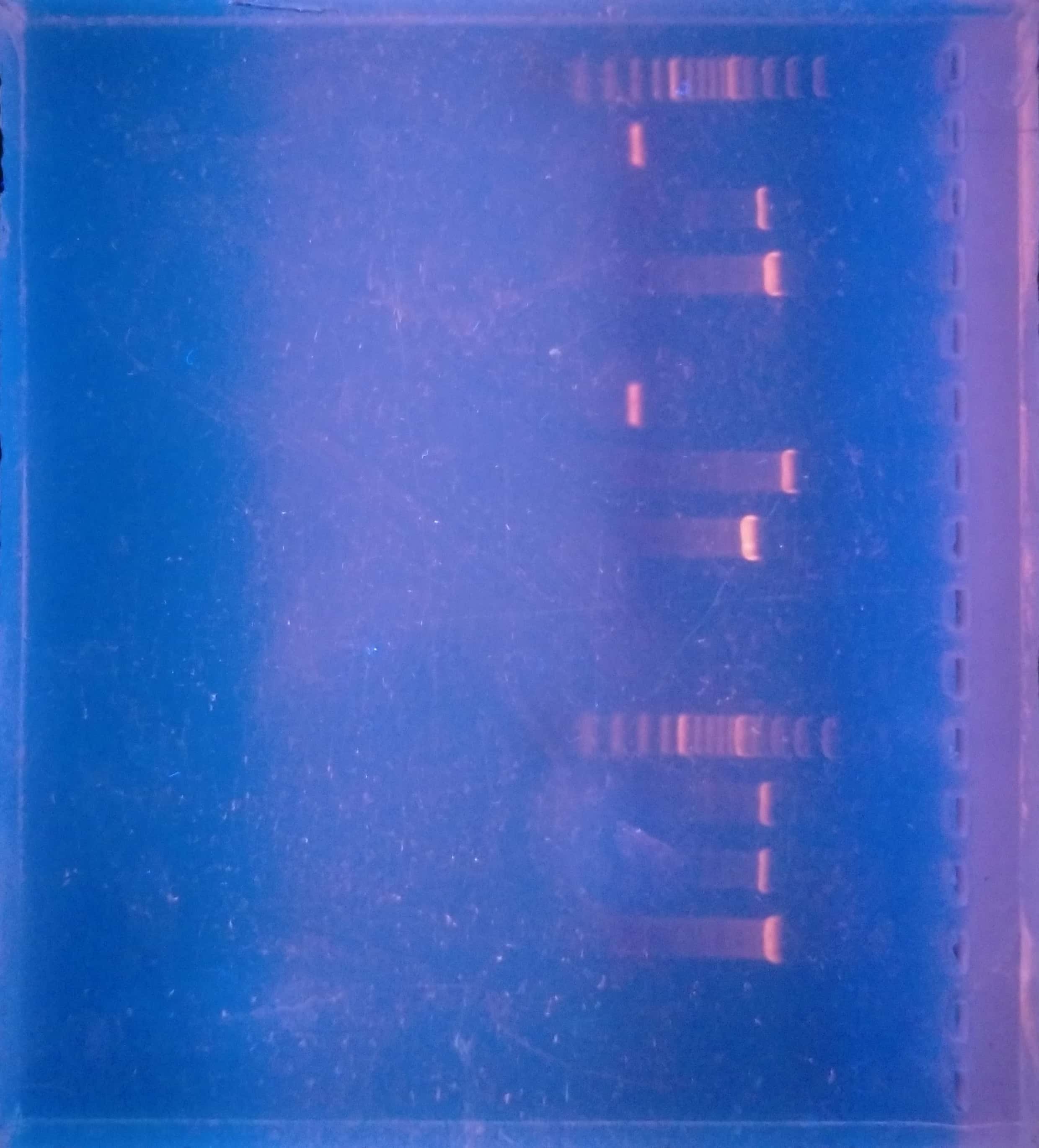


**1421 bp**

**1222 bp**

**M 1 2 3 4**

**298 bp**

1500

1000

300

100

**Supplementary Figure 3**. Gel electrophoresis analysis of PCR-amplified products demonstrating the removal of the ΩCm cassette, leaving a genomic scar. Lane 1: amplified PCR product of SG Δ*purB* showing the scar (298 bp). Lane 2: amplified PCR product of SG Δ*purB*:ΩCm (1222 bp). Lane 3: amplified PCR product of wild-type SG *purB* (1421 bp). Lane 4: negative control (no template)
